# Point‐of‐Care Biomarker Monitoring of Treatment Efficacy: Changes in Mouthrinse Active Matrix Metalloproteinase‐8 (aMMP‐8) Concentrations Following Periodontal Anti‐Infective Treatment

**DOI:** 10.1155/ijod/4285673

**Published:** 2026-02-24

**Authors:** Louis R. Gieselmann, Juulia Rintamarttunen, Mutlu Keskin, Ismo T. Räisänen, Taina Tervahartiala, Tommi Pätilä, Dirk Neefs, Andreas Pfützner, Timo Sorsa

**Affiliations:** ^1^ Department of Education and Psychology, Free University of Berlin, Berlin, Germany, fu-berlin.de; ^2^ Department of Oral and Maxillofacial Diseases, University of Helsinki, Helsinki, Finland, helsinki.fi; ^3^ Department of Periodontology, University of Kyrenia, Kyrenia, Cyprus, kyrenia.edu.tr; ^4^ Department of Oral and Maxillofacial Diseases, University of Helsinki and Helsinki University Hospital, Helsinki, Finland, helsinki.fi; ^5^ Department of Pediatric Surgery, New Children’s Hospital, University of Helsinki, Helsinki, Finland, helsinki.fi; ^6^ Department of Biomedical, Surgical, and Dental Science, Universitá degli Studii di Milano, Milan, Italy, unimib.it; ^7^ Department of Dentistry, University for Digital Technologies in Medicine and Dentistry, Wiltz, Luxembourg; ^8^ Pfützner Science and Health Institute, Mainz, Germany; ^9^ Institute for Internal Medicine and Laboratory Medicine, University for Digital Technologies in Medicine and Dentistry, Wiltz, Luxembourg; ^10^ Department of Dental Medicine, Karolinska Institutet, Stockholm, Sweden, ki.se

**Keywords:** aMMP-8, chairside test, diagnostic accuracy, matrix metalloproteinase-8 (MMP-8), mouthrinse sample, periodontal diagnosis, periodontal treatment, periodontitis, point-of-care testing, treatment monitoring

## Abstract

**Introduction:**

The active matrix metalloproteinase‐8 (aMMP‐8) is a functional biomarker of active periodontal tissue destruction. It bridges the gap between clinical findings and underlying biological processes, providing information on active collagen degradation that conventional clinical examinations do not capture.

**Methods:**

In a prospective within‐subject clinical study, we assessed point‐of‐care (PoC) mouthrinse aMMP‐8 concentrations, periodontal probing depth (PPD), and clinical attachment level (CAL) in 27 adults with stage III/IV (grade C) periodontitis before and 1 month after anti‐infective treatment. aMMP‐8 PoC results were validated by laboratory western blotting using an independent polyclonal MMP‐8 antibody.

**Results:**

At baseline, 85.2% of samples showed elevated aMMP‐8 PoC concentrations, declining to 18.5% post‐treatment. Baseline aMMP‐8 was significantly associated with disease severity, correlating with both PPD (*r*_*s*_ = 0.39, *p* = 0.045) and CAL (*r*_*s*_ = 0.39, *p* = 0.042). Following treatment, aMMP‐8 concentrations decreased significantly (*V* = 378, *p* < 0.001), with mean reductions of 78.5 ± 72.6 ng/mL (*d* = –1.08). Biochemical reductions paralleled clinical improvements, with 100% directional concordance between changes in aMMP‐8 and changes in PPD and CAL (95% CI 0.87–1.00). Reductions in aMMP‐8 were correlated with improvements in PPD (*r*_*s*_ = 0.47, *p* = 0.014) but not CAL (*r*_*s*_ = 0.12, *p* = 0.544). Linear regression analysis indicated that each 10 ng/mL reduction in aMMP‐8 corresponded to an estimated 0.034 mm gain in PPD.

**Conclusion:**

These findings demonstrate that aMMP‐8 PoC monitoring aligns with both baseline disease severity and short‐term periodontal healing. aMMP‐8 provides an objective biochemical dimension to periodontal assessment, capturing active collagen degradation and serving as a sensitive and clinically meaningful indicator of treatment response.

## 1. Introduction

Periodontitis is a common chronic inflammatory disease that results in the destruction of tooth attachment tissue [1]. It is the most common cause of tooth loss in adults [1] and is associated with several systemic diseases such as diabetes, cardiovascular disease, rheumatoid arthritis, COVID‐19 infections, cancer, Alzheimer’s disease, and depression [2–8]. To prevent periodontitis and the associated systemic risks of severe disease stages, early detection of disease progression is key [1, 9, 10].

Dysbiosis of the oral microbiota is a central risk to the development and progression of periodontitis. The progression of tissue destruction results from a nonphysiological host response to oral dysbiosis [1]. Collagen is the main structural protein in the periodontium [11] and matrix metalloproteinase (MMP)‐8 or neutrophil collagenase/collagenase‐2 is the most common collagen‐degrading protease in the diseased periodontium [12]. It has been extensively studied as a diagnostic biomarker for periodontitis in oral fluids (mouthrinse, saliva, gingival crevicular fluid, and peri‐implant crevicular fluid) [13]. MMP‐8 is primarily a neutrophil‐derived proenzyme that is activated by microbial virulence factors and/or host‐derived proinflammatory cytokines and reactive oxygen species [12, 14]. MMP‐8 is produced in the bone marrow during neutrophil development and is stored in its latent form in the intracellular granules of neutrophils. During the degranulation process, MMP‐8 is released extracellularly in inflammatory periodontal diseases, and its active form, active MMP‐8 (aMMP‐8), leads to active tissue destruction in periodontal diseases, reflected in increased aMMP‐8 concentrations in oral fluids [12, 14–18]. While the latent noncollagenolytic form of MMP‐8 is normally present in healthy humans, its active degranulating form, the collagenolytic aMMP‐8, increases in periodontal and peri‐implant inflammation, especially in the active phases of these diseases [15, 16, 19–25]. aMMP‐8 is a functional biomarker, that is, a metabolism‐based biomarker quantifying the activity of biological processes, distinguishing it from risk biomarkers, that is, biological indicators of disease antecedence like specific periodontal pathogens [26]. Thus, measuring aMMP‐8 compared to MMP‐8 improves the diagnostic accuracy in periodontitis management [12, 15, 16, 18, 20, 22]. Mouthrinse and gingival crevicular fluid/peri‐implant crevicular fluid, rather than saliva, are the most accurate oral fluid samples in aMMP‐8 diagnostics [12, 21, 27]. Further, aMMP‐8 is a functional biomarker of disease development but not a disease biomarker that necessarily correlates with the current clinical presentation of the patient [23, 28]. This is because in periodontitis, the process of enzymatic and proteolytic tissue degradation, i.e., the biological phenotype, precedes clinical signs and symptoms, i.e., the clinical phenotype [29]. Additionally, periodontitis progression is characterized by periods of exacerbation and remission [1]. Consequently, collagenolytic activity, reflected in aMMP‐8 concentrations, can be low in severe disease stages, most evidently following anti‐infective treatment [19, 23].

aMMP‐8 is positively associated with various parameters of periodontal disease, notably prospective radiographic bone loss (RBL) and clinical attachment loss [30–32]. The inhibition of aMMP‐8 through sub‐antimicrobial dose doxycycline (SDD) has been shown to lead to increased attachment gain during 2‐year periodontal maintenance compared to placebo controls [32–34], while increases in aMMP‐8 were associated with progressive attachment loss [32]. aMMP‐8 levels below the 20 ng/mL threshold have been reported in periodontally and systemically healthy individuals without prior experience or activity of periodontal disease and have been interpreted as a measure of periodontal and peri‐implant health [19, 35, 36].

Traditionally, diagnosis and monitoring of periodontal diseases rely on bleeding on probing, clinical attachment level measurement, probing depth, and radiographic examinations [1, 37–39]. However, longitudinal studies show that bleeding on probing alone is not a reliable predictor of periodontal tissue destruction in patients treated for periodontitis [40–42]. As Keskin et al. [39] noted, traditional periodontitis examinations and follow‐ups are time‐consuming. Additionally, mechanical probing can be painful for the patient, and repeated probing at periodontitis treatment follow‐up visits increases the likelihood of bacteremia [1, 39]. Measurements like BOP and periodontal probing depth (PPD) are influenced by the characteristics of the instrument used and the force applied by the researcher, which may lead to measurement error [41].

The current AAP/EFP classification system determines the stage of periodontitis based on measures of attachment loss [37, 41, 43]. To determine the grade of periodontitis activity, the system employs 5‐year clinical attachment level (CAL) or RBL [41]. Periodontitis is characterized by periods of exacerbation and remission and a nonlinear progression, while averaging CAL or RBL over 5 years to determine the risk of future progression assumes linearity [1, 10, 29]. Though periodontitis grade is recommended to be based on longitudinal evidence of CAL or RBL, the single‐time‐point bone loss/age ratio (% RBL/age) is more commonly used in clinical practice because it does not require longitudinal data [43]. Such indirect evidence of grade has been shown to lead to misclassifications compared to longitudinal evidence [44]. Bumm et al. [44] report grade A according to direct evidence was misclassified by indirect evidence in 100% of cases, grade B in 31%, and grade C in 43%. Generally, measures of attachment loss, for example, by radiological examination, provide retrograde information, i.e., occurred attachment loss and disease progression [1, 29, 45]. When the detection of active disease phases is based solely on retrograde information, that is, evidence of progressive CAL or RBL, treatment planning consequently is delayed by the time it takes for these clinical signs to manifest and be detected [10, 29].

Biomarkers like point‐of‐care (PoC) aMMP‐8 allow single‐time‐point assessments in a standardized, noninvasive, and time‐efficient way, making them a potentially useful tool in clinical routines [10, 29, 39, 45, 46]. Functional biomarkers have the characteristic of displaying physiological processes causal to disease progression, with aMMP‐8 measuring real‐time collagenolytic activity that is preceding attachment loss [15]. In consequence of its active role in the destructive pathophysiology leading to periodontitis, aMMP‐8 has been confirmed to be a useful PoC biomarker for the diagnosis and monitoring of periodontal disease activity by multiple studies [19, 21, 27, 31, 36, 38, 39, 47, 48]. Elevated levels of aMMP‐8 in oral fluid indicate active and ongoing periodontal disease and have been used to assess disease severity and treatment response [19, 31, 39, 47, 48]. aMMP‐8 PoC testing can aid in the diagnosis and classification of periodontal and peri‐implant diseases, particularly in determining the grade of disease progression [20, 21, 36–38, 41].

The 2017 World Workshop on the Classification of Periodontal and Peri‐Implant Diseases and Conditions has highlighted the potential of biomarker diagnostics for periodontology and future classification systems [10, 41]. PoC aMMP‐8 has been integrated into the staging and grading framework by Sorsa et al. [21] to indicate the grade of progression. Thus, aMMP‐8 may complement the current classification system as a grade modifier, such that patients with low or moderate progression risk according to clinical parameters but elevated aMMP‐8 should be considered at high risk of progression [21, 30–32]. While traditional clinical indicators of progression risk are retrograde and time‐lagged, aMMP‐8 concentrations, indicating real‐time physiological activities, can objectively indicate phases of disease exacerbation and remission characteristic of periodontal disease [1, 12, 18, 31, 32, 48].

The aim of this study was to evaluate the aMMP‐8 PoC test’s use in monitoring disease activity and treatment response in patients with severe periodontitis under anti‐infective treatment. Previous research has demonstrated a significant decrease in aMMP‐8 levels after periodontal treatment, indicating a decrease in active tissue destruction and clinical disease activity [12, 19, 35]. Keskin et al. [39] reported the diagnostic performance of aMMP‐8 PoC and immunofluorometric assay (IFMA) method in distinguishing periodontitis patients from healthy controls and monitoring treatment effects independent of smoking status. Using the treatment cohort from the same patient sample, this work extends these findings by quantitatively characterizing changes in aMMP‐8 in relation to PPD and CAL and by examining the correspondence between biochemical recovery and clinical healing outcomes.

## 2. Materials and Methods

### 2.1. Study Design and Population

**Figure 1:**
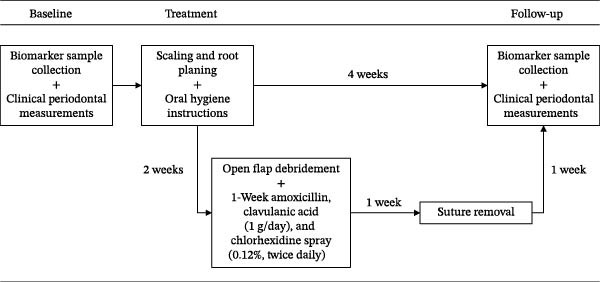
Study procedure. *Note:* Out of 27 patients, 7 (26%) required flap surgery and completed the additional treatment path.

The study employed a prospective within‐subject clinical study. The study protocol is presented in Figure 1. The study cohort consisted of a convenience sample of 27 Turkish periodontitis patients who applied for periodontitis treatment in a private clinic (Özel Fulya Ağız ve Diş Sağlığı Kliniği) in Tekirdağ, Turkey. The period of data collection was from 2021 to 2022. The study was approved by the Ethics Committee of Biruni University (2015‐KAEK‐71‐22‐06) and was conducted in accordance with the principles of the Declaration of Helsinki. All study participants gave verbal and written informed consent.

The inclusion criteria for the study were adult patients (≥18 years) with (i) interdental CAL ≥ 5 mm at the site of greatest loss, (ii) RBL of more than one‐third of the tooth root, (iii) number of teeth lost due to periodontitis ≤4 (Stage III periodontitis) and ≥5 (Stage IV periodontitis), and (iv) periodontitis progression category C. Patients were excluded from the study based on the following criteria: (i) AIDS (Acquired Immune Deficiency Syndrome), (ii) uncontrolled diabetes (HbA1c >7) and other chronic diseases related to immune system diseases such as Crohn’s disease, etc., (iii) pregnant and lactating patients, and (iv) patients who have undergone periodontitis treatment in the last year. The study involved 27 patients with periodontitis in risk category C, four of whom had stage III periodontitis and 23 who had stage IV periodontitis. The age of the patients was 30–70 years (mean = 47.3, SD = 11.9). The characteristics of the cohort are detailed in Table 1.

**Table 1:** Characteristics of study cohort.

Patient characteristics	
Age (in years)	47.3 ± 11.84

Gender	

Females	19 (70%)
Males	8 (30%)

Systemic status	
Healthy	22 (82%)
Cardiovascular diseases	2 (7%)
Hypothyroidism	3 (11%)

Medication	
No medication	9 (45%)
Levothyroxine sodium	3 (15%)
Beta‐1 selective blocker	2 (7%)
Atorvastatin	1 (4%)

Smoking (≥10 cigarettes a day, more than 5 years)	
Yes	13 (48%)
No	14 (52%)

Periodontitis stage	
Stage III	4 (17%)
Stage IV	23 (83%)

Periodontitis grade	
A	0 (0%)
B	0 (0%)
C	27 (100%)

### 2.2. Periodontal Examination Procedure

Clinical periodontal measurements of the patients were performed by M.K., an experienced specialist in periodontology, and assessed before periodontal treatment and 1 month after treatment (Figure 1). PPD and gingival margin level were measured on six surfaces of each patient’s tooth using a color‐coded Williams Michigan pocket gauge. CAL was calculated from PPD and the gingival margin level.

### 2.3. Periodontal Treatment Procedure

All patients were treated by M.K. and received anti‐infective periodontal treatment. The treatment procedure is presented in Figure 1. Nonsurgical treatment involved scaling and root planing to remove subgingival plaque and calculus, followed by oral hygiene instructions for self‐care. Two weeks after the nonsurgical periodontal treatment phase, surgical treatment followed for periodontal areas with irregular bone margin, alveolar ridge damage, or periodontal pockets that could not be properly treated by nonsurgical methods, such as in cases of furcation lesions of grade II–III, where treatment was intensified with surgical flaps to improve visibility. Open flap debridement was supported by a 1‐week course of antibiotics (amoxicillin and clavulanic acid 1 g/day) and a 1‐week course of chlorhexidine spray (0.12%, twice daily). Patients returned for suture removal 1 week after periodontal flap surgery.

### 2.4. Quantitative aMMP‐8 PoC Analysis

Concentrations of aMMP‐8 in patients’ mouthrinse were assessed chairside before treatment and 1 month after treatment using the aMMP‐8 PoC test (Periosafe, Dentognostics GmbH, Solingen, Germany) and a quantitative digital reader (ORALyzer, Dentognostics GmbH, Solingen, Germany). Mouthrinse samples were collected before clinical measurements according to the manufacturer’s instructions: Patients were instructed not to eat for 1 h before the testing. First, patients were asked to rinse their mouths with plain water (drinking water or distilled water) for 30 s and to spit out. After waiting for 1 min, they were instructed to rinse their mouth for 30 s with 5 mL of distilled water from the aMMP‐8 test kit (Periosafe) and spit the mouthrinse sample into the collection container. Using the test kits’ sterile syringe, 3–4 drops were removed from the container and dripped into the aMMP‐8 test cassette. The cassette was then immediately inserted into the ORALyzer digital reader, from which the quantitative results (aMMP‐8 ng/mL) were retrieved after 5 min of incubation. The remaining liquid in the container was transferred to Eppendorf tubes and stored at −70°C for further laboratory analysis.

### 2.5. Western Blot Procedure

The molecular forms of MMP‐8 were detected in mouthrinse samples using a modified enhanced chemiluminescence (ECL) western blotting protocol (GE Healthcare, Amersham, UK), as previously described by Rautava et al. [49]. Briefly, proteins from mouthrinse samples were separated by electrophoresis and electrotransferred onto nitrocellulose membranes (Protran, Whatman GmbH, Dassel, Germany). The membranes were incubated overnight with a monoclonal anti‐MMP‐8 primary antibody [50], followed by a horseradish peroxidase–conjugated secondary antibody (GE Healthcare, Buckinghamshire, UK) for 1 h. Membranes were washed four times in TBST (15 min each) between incubation steps. Protein bands were visualized using the ECL detection system according to the manufacturer’s instructions. Recombinant human MMP‐8 (100 ng; Calbiochem, Darmstadt, Germany) was used as a positive control for antibody specificity and band identification.

### 2.6. Statistical Analysis

The primary aim was to describe treatment‐related changes in aMMP‐8 and evaluate their correspondence with clinical improvements, assessed using directional concordance, correlation, and linear regression analyses. Because the dataset was fixed, a minimum detectable effect (MDE) analysis was performed to determine the smallest correlation that the available sample size (*n* = 27) could detect with 80% power at *α* = 0.05. Using the *pwr* package, the detectable effect size was *r* = 0.49 (*R* ^2^ = 0.24), indicating sensitivity to large effects.

aMMP‐8 values <10 ng/mL were coded as 7.07 ng/mL (limit of quantification divided by √2). Normality was assessed using the Shapiro–Wilk test and visual inspection of Q–Q plots. Dichotomous aMMP‐8 PoC results by cutoff (<20 vs. ≥20 ng/mL) at baseline and treatment follow‐up were summarized as counts and percentages. Descriptive statistics were calculated for aMMP‐8, PPD, and CAL as means and standard deviations, as well as corresponding within‐subject effect sizes (Cohen’s *d*_*p*_). For aMMP‐8, additional medians and interquartile ranges (IQR) were visualized due to skewed distribution.

Treatment‐induced changes in aMMP‐8 and clinical parameters were evaluated using paired *t*‐tests when change scores were normally distributed, and the Wilcoxon signed‐rank test when normality was violated. Directional concordance quantified the proportion of cases in which aMMP‐8 and the corresponding clinical variable changed in the same direction, with 95% binomial confidence intervals. Associations between aMMP‐8 and clinical parameters were evaluated using Pearson correlation for normally distributed variables and Spearman correlation when normality assumptions were not met. Unadjusted linear regression models were used to estimate the average change in PPD or CAL per 10 ng/mL decrease in aMMP‐8, providing a direct measure of biomarker‐clinical correspondence. Permutation tests with 5000 iterations were applied to assess the robustness of regression slopes without relying on parametric assumptions.

Exploratory classification analyses were conducted to examine whether aMMP‐8 change could discriminate clinical treatment response. For the purpose of this study, responder status was defined as achieving ≥1 mm improvement in the respective clinical parameter (PPD, CAL), consistent with prior work demonstrating that anti‐infective treatment yields short‐term improvements in these parameters of ~1 mm [51]. Logistic regression models were fitted, and discrimination of predicted probabilities was evaluated using receiver operating characteristic (ROC) analyses (AUC). Sensitivity, specificity, positive and negative predictive values (PPV, NPV), and the Matthews Correlation Coefficient (MCC) were computed using thresholds based on the Youden index.

All analyses were conducted in R version 4.4.1 (R Core Team, 2024). All figures were generated with *ggplot2*.

## 3. Results

**Figure 2:**
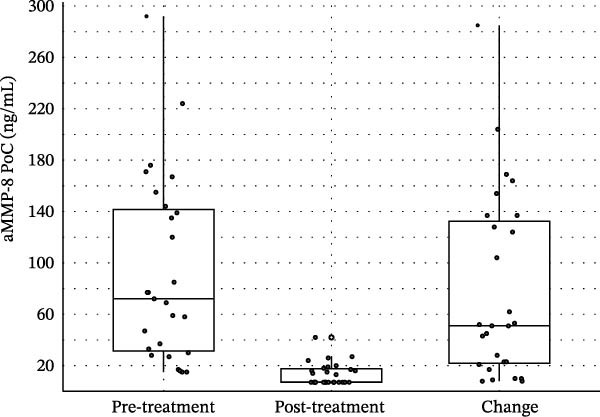
Boxplots of aMMP‐8 PoC concentrations.

At baseline, 85.2% of sites tested positive for aMMP‐8 (≥20 ng/mL), which decreased to 18.5% after treatment (Table 2). All participants showed a reduction in aMMP‐8 concentrations following treatment. Median aMMP‐8 concentrations declined markedly from 72.0 ng/mL (IQR = 31.5–141.5) to 7.1 ng/mL (IQR = 7.1–17.5) (Figure 2), corresponding to a mean reduction of 78.5 ± 72.6 ng/mL (*d*_*p*_ = –1.08) (Table 3). Visual inspection of Q–Q plots and Shapiro–Wilk tests confirmed non‐normality of both baseline aMMP‐8 (*W* = 0.892, *p* = 0.009) and aMMP‐8 change (*W* = 0.858, *p* = 0.002), supporting the use of nonparametric inference. The reduction in aMMP‐8 was statistically significant, as confirmed by the Wilcoxon signed‐rank test (*V* = 378, *p* < 0.001). Clinical parameters improved in parallel, with mean reductions of 1.5 ± 0.6 mm in PPD (*d*_*p*_ = –2.49, *t* (26) = 12.92, *p* < 0.001) and 1.2 ± 0.6 mm in CAL (*d*_*p*_ = –2.02, *t* (26) = 10.49, *p* < 0.001). Directional concordance of changes in aMMP‐8 with PPD and CAL was 100% (95% CI 0.87–1.00).

**Table 2:** Distribution of aMMP‐8 PoC results by cutoff before and after treatment.

		Pre‐treatment		Post‐treatment	
		Count	Percentage (%)	Count	Percentage (%)
aMMP‐8 PoC	Negative	4	14.8	22	81.5
	Positive	23	85.2	5	18.5

*Note:* The 20 ng/mL cutoff was applied to categorize samples as aMMP‐8 − (<20 ng/mL) or aMMP‐8 + (≥20 ng/mL).

**Table 3:** Treatment‐related changes in aMMP‐8 PoC concentrations and clinical parameters.

	Pre‐treatment		Post‐treatment		Change		Effect size
	*M*	SD	*M*	SD	*M*	SD	Cohen’s *d*_*p*_
aMMP‐8 PoC (ng/mL)	92.0	±72.2	13.6	±8.7	78.5	±72.6	−1.08
PPD (mm)	4.3	±0.6	2.8	±0.2	1.5	±0.6	−2.49
CAL (mm)	6.5	±1.9	5.3	±1.6	1.2	±0.6	−2.02

*Note*: aMMP‐8 values <10 ng/mL were coded as 7.07, i.e., LOQ/√2.

**Figure 3:**
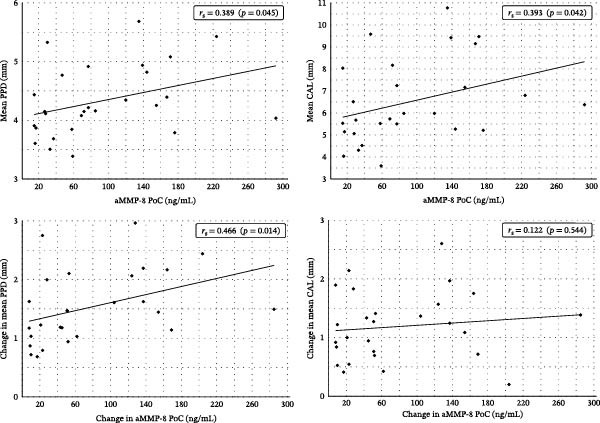
Spearman’s rank correlations between aMMP‐8 PoC concentrations and clinical parameters. *Note:* Baseline mean PPD and CAL were correlated with aMMP‐8 PoC concentrations (top). Treatment‐related changes in PPD and CAL were correlated with corresponding changes in aMMP‐8 PoC concentrations (bottom).

Spearman’s rank correlations revealed that baseline aMMP‐8 values were significantly positively associated with PPD (*r*_*s*_ = 0.39, *p* = 0.045) and CAL (*r*_*s*_ = 0.39, *p* = 0.042). Treatment‐related reductions in aMMP‐8 correlated significantly with improvements in PPD (*r*_*s*_ = 0.47, *p* = 0.014) but not with CAL (*r*_*s*_ = 0.12, *p* = 0.544) (Figure 3). Linear regression indicated that each 10 ng/mL decrease in aMMP‐8 was associated with a 0.034 mm improvement in PPD (95% CI 0.002–0.066, *p* = 0.037, *R* ^2^ = 0.16). The corresponding model for CAL was nonsignificant (*β* = 0.010 mm/10 ng/mL, 95% CI −0.023–0.043, *p* = 0.55, *R* ^2^ = 0.01). Permutation tests supported the significance of the PPD slope (*p* = 0.033) and confirmed the nonsignificance for CAL (*p* = 0.55).

**Figure 4:**
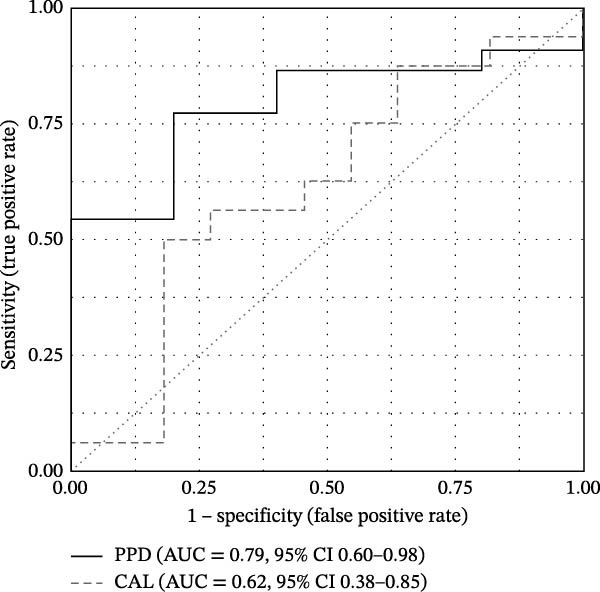
Receiver operating characteristic curve.

Exploratory analyses examined whether quantitative change in aMMP‐8 could classify clinical treatment response. Logistic regression models were fitted using aMMP‐8 change to predict responder status (≥1 mm improvement), and discrimination of the model‐derived predicted probabilities was evaluated using ROC analyses. For PPD, the ROC curve showed moderate discrimination with an AUC of 0.79 (95% CI 0.60–0.98), whereas discrimination for CAL was weaker (AUC = 0.62, 95% CI 0.38–0.85) (Figure 4). Thresholds based on the Youden index were used to compute sensitivity, specificity, PPV, NPV, and MCC, which are reported in Table 4.

**Table 4:** Performance of aMMP‐8 change for detecting clinical improvement.

	Change in aMMP‐8 PoC (ng/mL)				
	Sensitivity	Specificity	PPV	NPV	MCC
PPD improvement (≥1 mm)	0.77	0.80	0.94	0.44	0.47
CAL improvement (≥1 mm)	0.50	0.82	0.80	0.53	0.32

*Note:* Values are reported at the cutoff maximizing Youden’s J statistic, corresponding to an aMMP‐8 PoC reduction of ≥25.5 ng/mL for PPD improvement and ≥83.0 ng/mL for CAL improvement.

Laboratory western blot analysis confirmed the improvement in MMP‐8 activity independent of aMMP‐8 PoC testing. Representative western blot and aMMP‐8 PoC results collected before and after treatment are shown in Figure 5. In patient mouthrinse samples, MMP‐8 was detected in latent, active, and fragmented forms before treatment and showed a clear reduction in immunoreactivity after periodontal therapy, as analyzed with a polyclonal anti‐MMP‐8 antibody (Figure 5A).

Figure 5Representative western blot and aMMP‐8 PoC tests. *Note*: (A) Western blot analysis of the MMP‐8 enzyme and its various forms of mouthrinse samples with an independent polyclonal anti‐MMP‐8 antibody. Lane 1: Molecular weight standards 25–75 kDa, Lane 2: Recombinant human MMP‐8 (100 ng), Lane 3: Mouthrinse sample from a periodontitis patient before treatment, Lane 4: As in lane 3 but after anti‐infective treatment. pMMP‐8: pro‐MMP‐8; aMMP‐8: active MMP‐8; PMN: neutrophil‐type MMP‐8; Mes: MMP‐8 of mesenchymal cell type. (B) aMMP‐8 PoC tests. 1: post‐treatment result, one line, that is, negative (−) aMMP‐8 PoC test result, <20 ng/mL; 2: pre‐treatment result, two lines, that is, positive (+) aMMP‐8 PoC test result, ≥20ng/mL.

**Figure:**
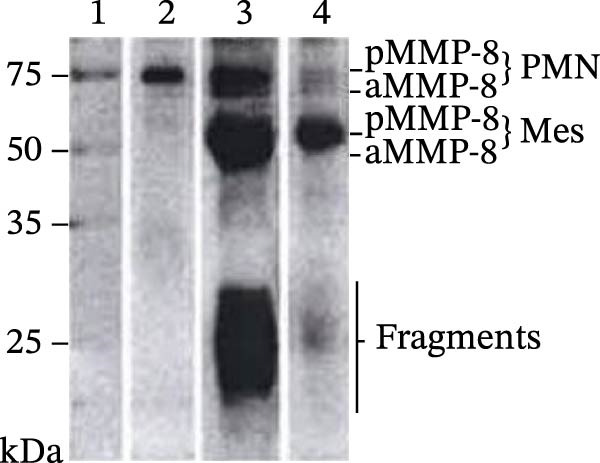
(A)

**Figure:**
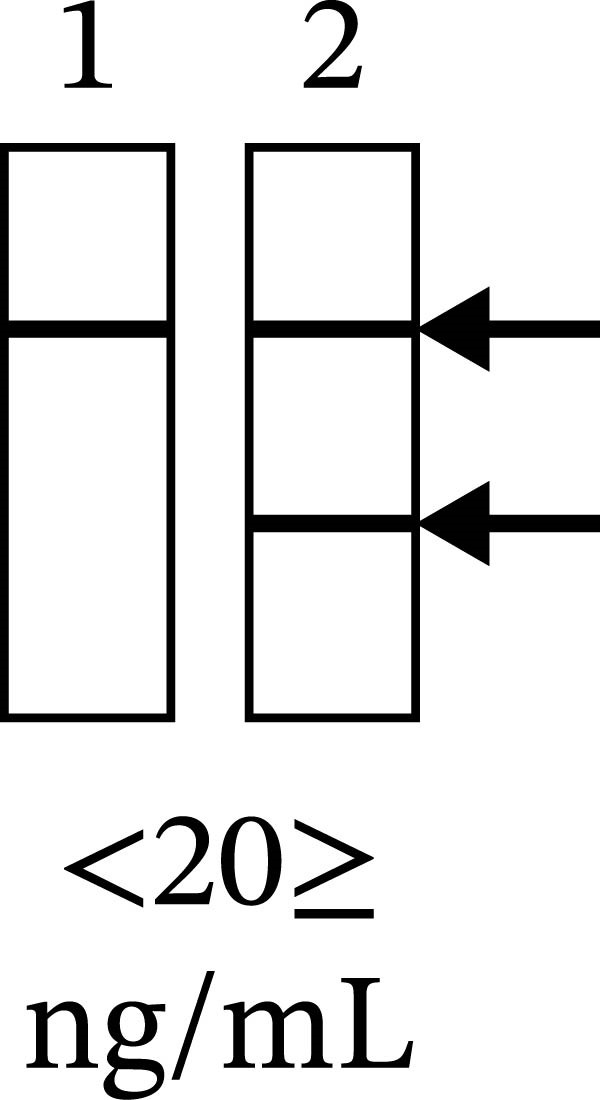
(B)

## 4. Discussion

This study investigated the correspondence between biochemical changes in aMMP‐8 and clinical improvements following periodontal anti‐infective treatment. At baseline, aMMP‐8 was positively associated with periodontitis severity (PPD, CAL). Following treatment, all patients exhibited decreases in aMMP‐8, indicating consistent suppression of collagenolytic activity. Anti‐infective treatment produced large reductions in both aMMP‐8 and clinical parameters. Greater decreases in aMMP‐8 were systematically aligned with greater PPD recovery, a relationship supported by correlation analysis, linear regression, and permutation testing. These findings support aMMP‐8 as a responsive and clinically relevant biomarker of short‐term periodontal healing and monitoring of treatment outcomes. The biochemical reduction of collagenolytic activity, as indicated by aMMP‐8, was confirmed by western blot analysis using an independent polyclonal antibody against MMP‐8. Exploratory analyses suggested that the magnitude of aMMP‐8 change may convey relevant information in classifying clinical treatment response, although these findings require confirmation in larger cohorts.

While broadly coherent with clinical indices, the aMMP‐8 biomarker adds important information about the biological phenotype, capturing an inherently different modality than the clinical phenotype [10, 29]. The aMMP‐8 biomarker, therefore, provides practitioners with important additional information that is absent when relying on clinical indices only. The presented results suggest that aMMP‐8 may be used as a relevant additional endpoint in periodontitis treatment monitoring and clinical studies [52, 53].

The finding of reduced collagenolytic activity following anti‐infective treatment is in line with previous studies showing decreases in aMMP‐8 and parallel clinical improvements in both periodontitis and peri‐implantitis [12, 19, 35, 54, 55]. As demonstrated by Keskin et al. [39] the cohort’s change in PoC aMMP‐8 and clinical parameters was independent of smoking status. Raivisto et al. [56] demonstrated how aMMP‐8 PoC screening and delivering prophylaxis and oral care instructions guided by the 20 ng/mL cutoff improved individuals at risk (≥20 ng/mL) to healthy aMMP‐8 levels after 1−2 intervention sessions. Thus, aMMP‐8 PoC testing has been employed in secondary and tertiary prevention of periodontitis and displayed a decrease in collagenolytic activity at treatment follow‐up, with the majority of patients reaching physiologically healthy levels [12, 19, 35, 54–57]. Besides the demonstrated application of monitoring treatment response, another potential of aMMP‐8 PoC analysis is the prediction of treatment response [20, 31, 48, 58]. There is evidence in the literature that elevated site‐specific aMMP‐8 concentrations at baseline are predictive of lower site‐specific CAL gain following anti‐infective treatment [48, 58] and higher bone loss following implant therapy [20, 31]. The systematic use of aMMP‐8 PoC testing can potentially serve practitioners to identify among patients those with further treatment need, and within these, to identify the sites at risk through site‐specific aMMP‐8 measurement [32, 48, 59, 60].

In the present study, not all participants reached below the 20 ng/mL threshold. Previous studies found that aMMP‐8 levels did not always reach the levels of healthy controls after 3 and 6 months of periodontal anti‐infective therapy [35]. The same applies to clinical parameters (PPD, CAL) after periodontal treatment [61]. aMMP‐8 release may still occur in periodontal areas that do not respond well to treatment [24, 35, 61]. Anti‐infective treatment of periodontitis is challenging, and variations in the initial level of aMMP‐8 activity and extent of reduction in aMMP‐8 levels are individual factors influencing the result at follow‐up, as well as characteristics of the practitioner and treatment [24, 61]. It is also important to consider, as Yilmaz et al. noted, that successful periodontal pocket healing depends on restoration of a symbiotic flora, resolution of inflammation, and subsequent reattachment and maturation of the supporting tissues. An incomplete achievement of any of these processes may lead to discrepancies between aMMP‐8 levels and clinical parameters [24].

A limitation of the present study is that the design had adequate statistical power to detect large effects, but was not suited to reliably detect moderate or small associations. Therefore, the nonsignificant association between changes in aMMP‐8 and CAL should be interpreted cautiously, as effects below this threshold could not be ruled out. The absence of a statistically significant association between changes in aMMP‐8 and CAL in this study contrasts with previous reports [30–32, 48, 58]. The observed data for CAL were inconclusive, as the 95% confidence interval for the regression effect was wide and spanned zero, indicating imprecision rather than evidence of no association. Thus, while a statistically significant result would have supported a true effect, the lack of significance should not be interpreted as confirming the absence of a relationship. Future investigations with larger samples can yield more stable estimates. Further, the relatively short follow‐up interval may have influenced the observed CAL outcomes. Structural tissue re‐attachment and maturation typically lag behind reductions in inflammatory activity, and longer follow‐up periods may be required to detect treatment‐related improvements in CAL that parallel biochemical changes. Increased follow‐up intervals are encouraged in future research investigating the relationship between CAL and aMMP‐8 change. A further limitation is that periodontitis grade classification relied on indirect evidence (%RBL/age) when direct evidence was unavailable. As stated in the introduction, indirect evidence has been shown to, at least in part, lead to misclassifications of periodontitis grade compared to direct evidence [44]. This may have introduced heterogeneity in progression grade at baseline.

PoC biomarker monitoring enables repeated, noninvasive assessment without mechanical probing or the associated risk of bacteremia. As the results of this study show, aMMP‐8 can index baseline PPD severity and treatment‐induced clinical improvements. These characteristics may be particularly useful in Steps 1 and 4 of periodontal therapy as outlined in the EFP S3 clinical practice guideline [52], which focus on (1) motivating patients for treatment and (4) maintaining outcomes through supportive periodontal care. Motivating patients to comply with treatment recommendations is challenging [52, 62, 63]. Patient adherence to recall intervals and oral hygiene instruction is essential for the success of supportive periodontal therapy [52, 64], yet adherence levels are frequently inadequate, as highlighted in the systematic review by Amerio et al. [65]. aMMP‐8 PoC testing constitutes biofeedback [62], which is an effective behavior‐change intervention [62, 63, 66–68]. Feedback, by definition, provides information on goal progress [69]. In terms of Control Theory, aMMP‐8 provides information on a current and goal state, and their discrepancy, that is, the observed aMMP‐8 concentration in relation to the 20 ng/mL cutoff [70, 71]. Minimizing this discrepancy becomes an actionable, proximal goal shared between patient and practitioner [68, 70–75]. Negative feedback, i.e., a perceived discrepancy to one’s goal, shifts the locus of attention [76]. Consequently, an elevated aMMP‐8 value in screening or a positive aMMP‐8 change score in monitoring presumably draws attention to a biological discrepancy. Such biofeedback about increased collagenolytic breakdown and corresponding goal discrepancy may motivate patients to minimize it [67, 70, 77–79]. Consequently, biofeedback on collagenolytic activity may serve as a communicative tool to convey the importance of oral hygiene instructions and explain treatment needs to patients [9, 52, 62]. As aMMP‐8 can be analyzed in 5 min at the PoC, it can serve practitioners to easily and time‐efficiently communicate currently active periodontal degeneration. Further, treatment efficacy can be indicated by aMMP‐8 PoC testing [12, 19, 35, 54, 55]. The aMMP‐8 PoC biomarker may therefore be used as a practical periodontal monitoring and motivational feedback system. In this study, all patients improved in aMMP‐8 following treatment, but not all reached below the test’s cutoff. Importantly, when post‐treatment aMMP‐8 values remain ≥20 ng/mL but show substantial improvement from baseline, this still indicates goal progress to patients. Thus, both the dichotomous result based on the 20 ng/mL cutoff and the change score may support motivational communication [45, 62, 67, 68, 77], helping patients recognize the benefits of their efforts. Future research should examine the motivational and communicative effects of biomarker‐based feedback in periodontal care, including whether aMMP‐8 monitoring can improve adherence and long‐term treatment outcomes.

## 5. Conclusions

aMMP‐8 concentrations in mouthrinse reflected baseline disease severity and decreased consistently after anti‐infective treatment. All patients showed biochemical improvement, and the magnitude of aMMP‐8 reduction closely paralleled clinical recovery. Reductions in aMMP‐8 were systematically aligned with improvements in PPD, supported by correlation analysis, linear regression, and permutation testing. Directional concordance between biochemical and clinical changes was complete. CAL improved clinically but showed no detectable association with aMMP‐8 change within the short follow‐up interval and available sample precision. Taken together, these findings indicate that aMMP‐8 is a responsive and clinically relevant biochemical marker of short‐term periodontal healing. It captures ongoing collagenolytic activity that is not represented by clinical indices alone and therefore provides complementary information on the biological phenotype of disease resolution. The data support the use of aMMP‐8 PoC testing as an adjunctive endpoint in monitoring treatment outcomes. Larger and longer‐term studies are warranted to determine how incorporating aMMP‐8 into routine monitoring influences long‐term periodontal stability and clinical decision‐making.

## Author Contributions

Study concept: Juulia Rintamarttunen, Mutlu Keskin, Ismo T. Räisänen, Taina Tervahartiala, Tommi Pätilä, and Timo Sorsa. Study design: Juulia Rintamarttunen, Mutlu Keskin, Ismo T. Räisänen, Tommi Pätilä, Dirk Neefs, Andreas Pfützner, and Timo Sorsa. Data acquisition: Juulia Rintamarttunen, Mutlu Keskin, Ismo T. Räisänen, Tommi Pätilä, and Taina Tervahartiala. Quality control of data and algorithms: Louis R. Gieselmann, Juulia Rintamarttunen, Mutlu Keskin, Ismo T. Räisänen, Dirk Neefs, Andreas Pfützner, and Taina Tervahartiala. Data analysis and interpretation: Louis R. Gieselmann, Juulia Rintamarttunen, Mutlu Keskin, Ismo T. Räisänen, Dirk Neefs, Andreas Pfützner, and Tommi Pätilä. Manuscript preparation: Louis R. Gieselmann, Juulia Rintamarttunen, Mutlu Keskin, Ismo T. Räisänen, Tommi Pätilä, Dirk Neefs, Andreas Pfützner, and Timo Sorsa. Manuscript editing: Louis R. Gieselmann, Juulia Rintamarttunen, Mutlu Keskin, Ismo T. Räisänen, Tommi Pätilä, Dirk Neefs, Andreas Pfützner, Taina Tervahartiala, and Timo Sorsa. Manuscript review: Louis R. Gieselmann, Juulia Rintamarttunen, Mutlu Keskin, Ismo T. Räisänen, Taina Tervahartiala, Tommi Pätilä, Dirk Neefs, Andreas Pfützner, and Timo Sorsa.

## Funding

Juulia Rintamarttunen received financial support for this study from the Finnish Dental Society Apollonia, Finland, Grant Number: 20240010. Timo Sorsa received financial support for this study from the Finnish Dental Society Apollonia, Finland; the Karolinska Institute, Stockholm, Sweden; the Helsinki and Uusimaa Hospital District (HUS), Grant Numbers: Y1014SULE1, Y1014SL018, Y1014SL017, TYH2019319, TYH2018229, TYH2017251, TYH2016251, and TYH2022225. The funders had no role in the design of the study; in the collection, analyses, or interpretation of data; in the writing of the manuscript, or in the decision to publish the results. Open access publishing facilitated by Helsingin yliopisto, as part of the Wiley ‐ FinELib agreement.

## Disclosure

All authors have read and agreed to the published version of the manuscript.

## Ethics Statement

The protocol of this study was approved by the Biruni University Ethics Committee (2015‐KAEK‐71‐22‐06) and was conducted in accordance with the Helsinki principles.

## Consent

Written acceptance of informed consent was obtained from all subjects involved in the study.

## Conflicts of Interest

Timo Sorsa is the inventor of U.S. patents 1,274,416, 5,652,223, 5,736,341, 5,864,632, 6,143,476 and US 2017/0023571A1 (issued June 6, 2019), WO 2018/060553 A1 (issued May 31, 2018), 10,488,415 B2, and US 2017/0023671A1, Japanese Patent 2016‐554676 and South Korean Patent No. 10‐2016‐7025378. Louis R. Gieselmann is employed part‐time as Knowledge Manager at Dentognostics GmbH. This role involves tasks unrelated to the study, and Dentognostics GmbH had no role in the study design, data collection, analysis, interpretation, or manuscript preparation. All other authors declare no conflicts of interest.

## Data Availability

The data that support the findings of this study are available from the corresponding author upon reasonable request.
